# Dietary Carrageenan Amplifies the Inflammatory Profile, but not Permeability, of Intestinal Epithelial Cells from Patients With Crohn’s Disease

**DOI:** 10.1093/ibd/izae306

**Published:** 2024-12-24

**Authors:** Eva Vissers, Judith Wellens, Lorenzo Giorio, Ward Zadora, Bram Verstockt, Marc Ferrante, Séverine Vermeire, Christophe Matthys, Kaline Arnauts, João Sabino

**Affiliations:** Department of Chronic Diseases and Metabolism (CHROMETA), Translational Research Center for Gastrointestinal Disorders (TARGID), KU Leuven, Herestraat 49, 3000 Leuven, Belgium; Department of Chronic Diseases and Metabolism (CHROMETA), Translational Research Center for Gastrointestinal Disorders (TARGID), KU Leuven, Herestraat 49, 3000 Leuven, Belgium; Department of Gastroenterology and Hepatology, UZ Leuven, Herestraat 49, 3000 Leuven, Belgium; Department of Chronic Diseases and Metabolism (CHROMETA), Translational Research Center for Gastrointestinal Disorders (TARGID), KU Leuven, Herestraat 49, 3000 Leuven, Belgium; Department of Microbiology, Immunology and Transplantation, Nephrology and Renal Transplantation Research Group, KU Leuven, Herestraat 49, 3000 Leuven, Belgium; Department of Chronic Diseases and Metabolism (CHROMETA), Translational Research Center for Gastrointestinal Disorders (TARGID), KU Leuven, Herestraat 49, 3000 Leuven, Belgium; Department of Gastroenterology and Hepatology, UZ Leuven, Herestraat 49, 3000 Leuven, Belgium; Department of Chronic Diseases and Metabolism (CHROMETA), Translational Research Center for Gastrointestinal Disorders (TARGID), KU Leuven, Herestraat 49, 3000 Leuven, Belgium; Department of Gastroenterology and Hepatology, UZ Leuven, Herestraat 49, 3000 Leuven, Belgium; Department of Chronic Diseases and Metabolism (CHROMETA), Translational Research Center for Gastrointestinal Disorders (TARGID), KU Leuven, Herestraat 49, 3000 Leuven, Belgium; Department of Gastroenterology and Hepatology, UZ Leuven, Herestraat 49, 3000 Leuven, Belgium; Department of Chronic Diseases and Metabolism, Clinical and Experimental Endocrinology, KU Leuven, Herestraat 49, 3000 Leuven, Belgium; Department of Endocrinology, UZ Leuven, Herestraat 49, 3000 Leuven, Belgium; Department of Chronic Diseases and Metabolism (CHROMETA), Translational Research Center for Gastrointestinal Disorders (TARGID), KU Leuven, Herestraat 49, 3000 Leuven, Belgium; Department of Chronic Diseases and Metabolism (CHROMETA), Translational Research Center for Gastrointestinal Disorders (TARGID), KU Leuven, Herestraat 49, 3000 Leuven, Belgium; Department of Gastroenterology and Hepatology, UZ Leuven, Herestraat 49, 3000 Leuven, Belgium

**Keywords:** ultra-processed foods, emulsifiers, intestinal epithelium, inflammatory bowel diseases, organoids

## Abstract

**Background:**

The consumption of ultra-processed foods has increased significantly worldwide and is associated with the rise in inflammatory bowel diseases. However, any causative factors and their underlying mechanisms are yet to be identified. This study aimed to further elucidate whether different types of the dietary emulsifier carrageenan (CGN) can alter the permeability and inflammatory state of the intestinal epithelium.

**Methods:**

Caco-2/HT29-MTX cocultures (*n* = 4) were exposed to either κ-, ι-, or λ-CGN (100 µg mL^–1^) for 24 hours. Organoid-derived monolayers from patients with Crohn’s Disease (CD) were exposed to κ-CGN (100 µg mL^–1^) for 48 hours (*n* = 10). In both models, an inflamed condition was established by adding a mix of inflammatory stimuli. Changes in permeability were measured by transepithelial electrical resistance (TEER). In the organoid-derived monolayers, cytokines were quantified in the apical and basolateral supernatant and gene expression was analyzed with RT-qPCR.

**Results:**

None of the CGN subtypes altered permeability of non-inflamed or inflamed Caco-2/HT29-MTX cocultures. In organoid-derived monolayers, κ-CGN did not affect TEER, but induced alterations in the gene expression of tight junctions and mucus proteins. Expression of *TNF*, *IL8*, and *IL1B* increased upon κ-CGN stimulation, both in inflamed and non-inflamed monolayers. Cytokine release in the supernatant was increased by κ-CGN for IL-6, IL-13, IL-4, IL-2, and IL-10.

**Conclusions:**

Dietary CGN caused upregulation of inflammatory markers and affected cytokine release of intestinal epithelial cells from CD patients, while permeability remained unaltered. When inflammation was already present, this pro-inflammatory effect was more pronounced, suggesting a role for dietary CGN during active CD.

## Graphical Abstract

**Graphical Abstract:**
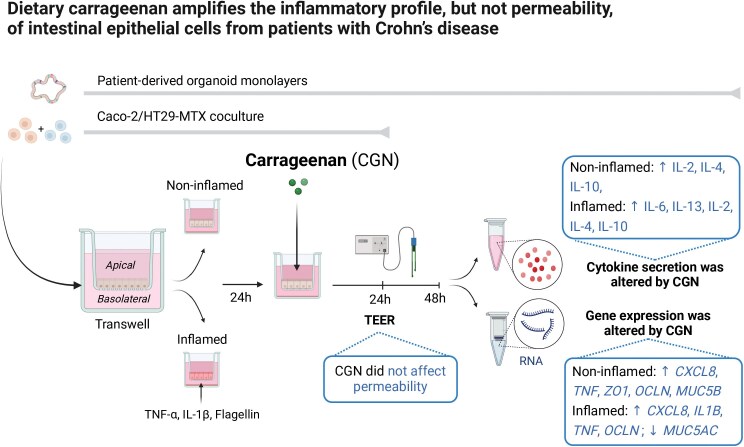

**Key Messages**

**What Is Already Known?**

- Dietary carrageenan is hypothesized to increase epithelial permeability and induce intestinal inflammation, but studies showed conflicting results and its effect on the intestinal epithelium remains unclear.

**What Is New Here?**

- A head-to-head comparison of different types of carrageenan showed that none of the subtypes directly altered permeability of colonic epithelial cell lines. On top, the use of a patient-derived epithelial model showed that carrageenan alters the inflammatory profile and that this is more pronounced in the presence of inflammation, as during active Crohn’s disease.

**How Can This Study Help Patient Care?**

- This study provides insight into the effects of carrageenan on the inflamed and non-inflamed intestinal epithelium, contributing to more targeted dietary guidelines for patients with inflammatory bowel diseases.

## Introduction

Carrageenan (CGN) is a commonly used food additive (E407) with properties as a gelling, thickening, and stabilizing agent.^1^ It is obtained from red seaweeds and consists of high-molecular-weight sulfated polygalactans. The 3 major types used in the food industry are kappa- (κ-), iota- (ι-), and lambda- (λ-) CGN, which differ in the number and position of sulfate groups. In Europe, CGN is authorized at *quantum satis* levels (ie, the amount needed to achieve the desired result) in almost all foods, except for some foods with a maximum permitted level (eg, infant formula: 300 mg kg^-1^).^1,2^ The main dietary sources of CGN are processed foods such as dairy products, confectionery, and processed meat products.^1^ The European Food Safety Authority estimated the average intake of CGN to be 5.0–88.9 mg kg^-1^ body weight per day in adults.^1^

Already in the 1970s, the safety of different types of CGN has been questioned. In many studies, CGN was used to induce inflammation by injection in animal paws or lung cavities.^3^ Also in the gastrointestinal tract of rodents, CGN was shown to induce ulcerative lesions, comparable to human ulcerative colitis (UC).^4^ However, the CGN used in these colitis models was a degraded form, so-called poligeenan. Food-grade CGN has an average molecular weight of 200-400 kDa, while poligeenan has a molecular weight of 10-20 kDa. Notably, degraded CGN is not used in the food industry and there is no evidence that food-grade CGN gets degraded in the gastrointestinal tract.^5^

More recently, a growing body of research has incriminated food-grade CGN as well, suggesting a role for CGN in inflammatory bowel diseases (IBD).^6,7^ Inflammatory bowel diseases comprise 2 main entities, Crohn’s disease (CD) and UC. Both are characterized by chronic inflammation in the gastrointestinal tract. Although the exact pathogenesis of IBD remains unclear, genetic susceptibility, microbial dysbiosis, unbalanced immune responses, and environmental factors are known to be involved. Epidemiological studies have shown associations between the consumption of ultra-processed foods (UPFs) and IBD.^8^ Ultra-processed foods are increasingly consumed worldwide and can be defined as food products that go through a series of industrial processes and consist of substances derived from foods and food additives.^9^ Since UPFs often contain dietary emulsifiers and thickeners, it is hypothesized that emulsifiers, including CGN, play a role in the increasing prevalence of IBD.^10^ Moreover, it was shown that patients with IBD consume significantly more emulsifiers compared to healthy individuals.^11^

To date, it is still unclear if CGN is involved in the development and disease course of IBD. Preclinical studies using colonic epithelial cell lines and animal models proposed that CGN can directly increase intestinal permeability and promote inflammation.^12–15^ Nevertheless, these results could not be replicated by other research groups.^16^ Moreover, the interaction between the intestinal microbiota and dietary CGN is not completely understood. Several animal studies have proposed that CGN can modulate the gut microbiota, creating a pro-inflammatory environment,^17–19^ while others state that CGN remains unaltered throughout the whole gastrointestinal tract.^20,21^ Human trials with CGN are very limited and 2 recent trials with UC patients also showed conflicting results.^22,23^ Intervention trials with CD patients are still lacking.

Given the conflicting data concerning the effect of CGN on intestinal permeability and inflammation and its role in IBD, we here aimed to explore the direct effect of dietary CGN on the intestinal epithelium. Therefore, we used an organoid-derived Transwell model originating from IBD patients as we hypothesize that patients with IBD are more sensitive to intestinal barrier disruption. Since the involvement of dietary factors is well established for CD, while for UC this connection is less clear, we focused in our experiments on CD.^24^

## Materials and Methods

**Figure 1.**
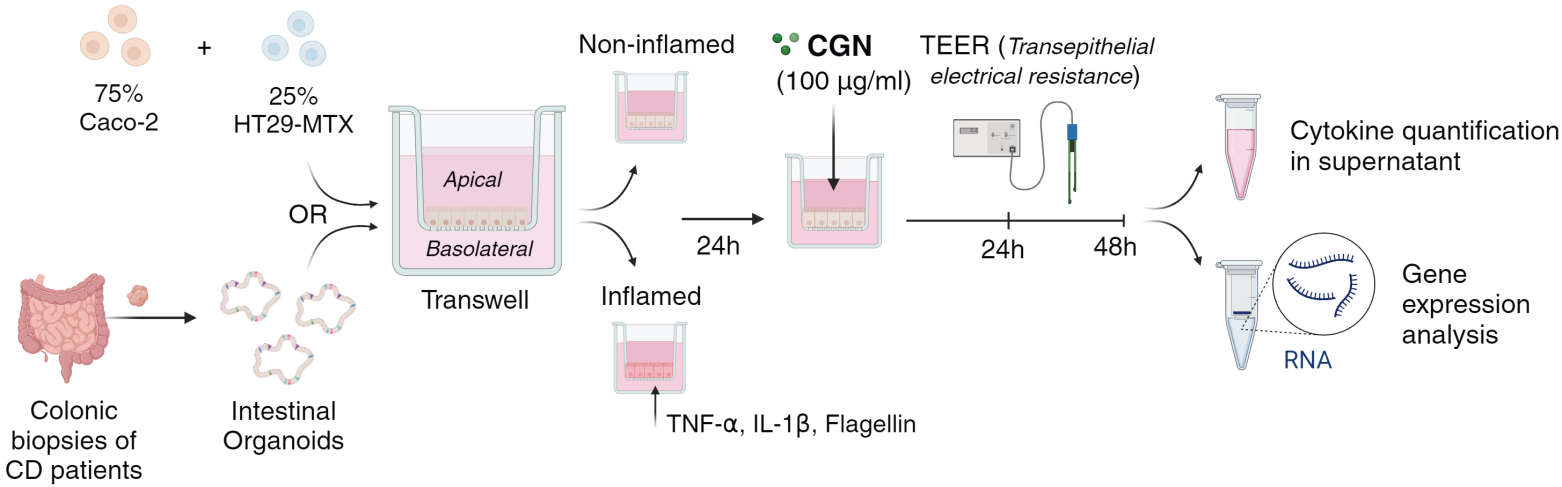
Overview of the experimental setup. Caco-2/HT29-MTX cocultures were established by seeding 75% Caco-2 cells and 25% HT29-MTX cells on Transwell inserts. After a differentiation period of 20 days, inflammation was induced. On day 21, the apical side of the epithelial cells was stimulated with 100 µg mL^-1^ of κ-, ι-, or λ-carrageenan (CGN), and the basolateral side was renewed with regular culture medium or the inflammatory mix. Transepithelial electrical resistance (TEER) was measured during the next 24 hours. Organoid-derived monolayers were derived from 5 patients with Crohn’s disease. Organoids were seeded on Transwells and inflammation was induced when a confluent monolayer was formed (day 6). The next day, organoid-derived monolayers were stimulated with 100 µg mL^-1^ κ-CGN and TEER was measured. After 48 hours, the cells were collected for RNA expression analysis and supernatant was collected for the quantification of cytokines. Figure created with Biorender.com.

An overview of the experimental setup is displayed in Figure 1.

### Cell Culture

Caco-2 cells were obtained from the American Type Culture Collection (ATCC, NCI-PBCF-HTB37) at passage 2. HT29 cells, treated with methotrexate (HT29-MTX), were obtained from the European Collection of Authenticated Cell Cultures (ECACC, HT29-MTX-E12, 12040401) at passage 50. Both cell lines were cultured separately in Dulbecco’s modified Eagle’s medium (DMEM) with high glucose (Gibco, 11965092), supplemented with 10% Fetal Bovine Serum (FBS) (Heat inactivated, Biowest, S181H), 1% nonessential amino acids (NEAA) (Gibco, 11140050) and 100 U mL^-1^ penicillin, 100 µg mL^-1^ streptomycin solution (Gibco, 15140122). Culture medium was replaced every 2-3 days and cells were maintained at 37 °C, 5% CO_2_. Every 5-7 days, cells were passaged with the use of 0.25% trypsin/ethylenediamine tetraacetic acid (EDTA) (Gibco, 25200056). Caco-2 and HT29-MTX cells were used for experiments between passages 5 and 25 and passages 55 and 75, respectively.

### Coculture of Caco-2 and HT29-MTX Cells on Transwell Inserts

Caco-2 and HT29-MTX cells were harvested, resuspended to a concentration of 2.6 × 10^5^ cells cm^-2^, and seeded on 0.33 cm^2^ Transwell inserts (0.4 µm pore size, polyester membrane, 3470, Corning Costar) at a ratio of 75% Caco-2 cells and 25% HT29-MTX cells. Apical and basolateral media were replaced every 2-3 days. After the acquired differentiation period (21 days), inflammation was induced if required (see “Induction of Inflammation” section), and cocultures were stimulated with CGN (see “Carrageenan Exposure” section) at the apical side. Integrity of the cell layers was checked before the experiment by measuring the transepithelial electrical resistance (TEER). By day 20, Caco-2/HT29-MTX cocultures reached a mean TEER value of 1087.4 Ω × cm² (min: 986.2 Ω × cm²; max: 1199.3 Ω × cm²).

### Human Biopsy Collection

Mucosal biopsies from non-inflamed colon segments of 5 patients with CD were obtained during routine endoscopy, following informed consent (S53684, approved by the Ethics Committee of the University Hospitals Leuven). Patient characteristics are shown in Table 1. Biopsies were collected in cold basal medium (BM) and processed within 2 hours. Basal medium consisted of DMEM:F12 (Gibco, 12634010) supplemented with 1× GlutaMax (Gibco, 35050038), 10 mM HEPES (Gibco, 15630056) and 100 U mL^-1^ penicillin, 100 µg mL^-1^ streptomycin (Gibco, 15140122).

**Table 1.** Baseline characteristics of CD patients (*n* = 5).

Baseline characteristics	Numbers
Male/female (%)	2/3 (40/60)
Age at endoscopy: median (IQR)	46.92 (34.87-48.32)
Disease duration at endoscopy (years): median (IQR)	21.49 (14.45-25.25)
SES-CD score: median (IQR)	10.5 (7.5-15.75)
Therapy at the moment of endoscopy:	
Biologicals (%)	3 (60) (1 ustekinumab, 2 risankizumab)
None (%)	2 (40)

Abbreviations: CD, Crohn’s disease; IQR, interquartile range; SES-CD, Simple Endoscopic Score for Crohn’s Disease.

### Intestinal Crypt Isolation and Organoid Culture

Intestinal crypts were isolated from 4 colonic biopsies per patient as described before.^25–27^ In short, colonic biopsies were washed thoroughly in chelating solution (5.6 mM Na_2_HPO_4_, 8.0 mM KH_2_PO_4_, 96.2 mM NaCl, 1.6 mM KCl, 43.4 mM sucrose, 54.9 mM d-sorbitol, 0.5 mM dl-dithiothreitol in deionized water) and incubated for 45 min in chelation solution supplemented with 2 mM EDTA at 4 °C on a rocking platform. Thereafter, biopsies were disrupted by rigorously pipetting with chelation solution to loosen and collect the crypts. The crypts were resuspended in 50% Matrigel (phenol red free, growth factor reduced, Corning, 356231) diluted by BM, and 4 droplets of 10 µL were plated in every well of a 24-well tissue culture plate. The culture plates were put at 37 °C for at least 20 min to allow Matrigel polymerization. Next, human expansion medium (HM) (BM supplemented with growth factors, Table S1) was added. The medium was replaced every other day and the organoids were split mechanically every 5-7 days, depending on their growth rate.

### Intestinal Organoid-Derived Monolayers

Organoid-derived monolayers were cultured as described before.^27^ Colonic organoid cultures were expanded and transferred to Transwell inserts (0.4 µm pore size, polyester membrane, 3470, Corning Costar), 3-5 days after splitting. For each patient, 2 independent experiments were performed (*n* = 10). Transwell inserts were coated with 0.1 mg mL^-1^ collagen type I (rat tail, Corning, 354236) diluted in 0.02 M acetic acid for 24 h at 37 °C. The next day, Transwells were washed twice with phosphate-buffered saline (PBS) (Gibco, 10010023) and pre-incubated with 100 µL of 50% HM, diluted with BM and supplemented with 10 µM Rho-associated kinase inhibitor (ROCKi) (Y-27632, Tocris Bioscience and R&D Systems). For each Transwell insert, 3 wells with organoids were collected with ice-cold BM and mechanically dissociated. Next, organoids were treated with 0.5 mM EDTA in PBS and centrifuged for 5 min at 350 *g*. The supernatant was removed and the pellet was incubated with 0.25% Trypsin/EDTA for 5 min at 37 °C. During the dissociation process, cells were regularly checked under a light microscope and if necessary, further mechanically dissociated by pipetting until only small clumps of cells remained. Trypsin was inactivated using BM with 10% FBS and the cells were centrifuged for 5 min at 350 *g*. The pellet was resuspended in 50% HM + ROCKi and 100 µL cell suspension was seeded in the apical compartment of each Transwell. The basolateral compartment was filled with 600 µL of 50% HM + ROCKi. After 24 hours, monolayers were carefully rinsed with PBS and the medium was replaced with 50 % HM without ROCKi. Apical and basolateral media was refreshed every other day until confluency (approximately 5-7 days) as monitored by TEER measurements. Organoid-derived monolayers reached a mean TEER value of 3175.8 Ω × cm² by day 6 (min: 2340.1 Ω × cm²; max: 4127.7 Ω × cm²).

### Induction of Inflammation

To mimic an IBD-like inflammatory phenotype, inflammation was induced in Caco-2/HT29-MTX cocultures and organoid-derived monolayers, 24 hours prior to CGN exposure (Figure 1). When confluent monolayers were formed, the basolateral side of the cells was stimulated with control medium (CTRL) or a combination of inflammatory cytokines diluted in culture medium (supplemented DMEM for Caco-2/HT29-MTX cocultures and 50% HM for organoid-derived monolayers) (INFL). This inflammatory mixture was previously defined by Arnauts et al.^26^ and contained 100 ng mL^-1^ TNF-α (Invivogen, rcyc-htnfa), 20 ng mL^-1^ IL-1β (Peprotech, 200-01B) and 1 µg mL^-1^ Flagellin (Invivogen, tlrl-stfla). Starting CGN exposure, the inflammatory mixture was renewed and maintained throughout the full experiment.

### Carrageenan Exposure

The following types of food-grade CGN were obtained from Sigma-Aldrich: κ-CGN (22048), ι-CGN (C1138), and λ-CGN (22049). Carrageenan was dissolved to a concentration of 100 µg mL^-1^ in regular culture medium (supplemented DMEM for Caco-2/HT29-MTX cocultures and 50% HM for organoid-derived monolayers). This concentration is believed to fall within the physiological range, as Borthakur et al. previously estimated the concentration of CGN in the intestinal lumen to be ±67 µg mL^-1^.^12^ On the day of the experiment (day 21 for Caco-2/HT29-MTX cocultures and day 7 for organoid-derived monolayers), the apical media was replaced by media supplemented with CGN (Figure 1). In the control conditions, the apical media was renewed with regular culture medium, and the basolateral media was renewed with regular culture medium (CTRL) or the inflammatory mixture (INFL).

### Transepithelial Electrical Resistance Measurements

The permeability of cell monolayers was determined by measuring Transepithelial Electrical Resistance (TEER), using an EVOM3 epithelial Volt/Ohm meter and STX4 electrodes (World Precision Instruments). Transwells were taken out of the incubator 15-20 minutes prior to the measurement to allow temperature stabilization. The electrodes were rinsed in 70% ethanol followed by PBS and 2 measurements per Transwell were performed. Transepithelial Electrical Resistance values are corrected for the surface area of the Transwells (0.33 cm²) and the resistance of a blank Transwell, and are expressed as the percentual change compared to baseline (= measurement right before stimulation with CGN (t0)).

### RNA Extraction

Organoid-derived monolayers were washed twice with PBS and incubated with 0.25% Trypsin-EDTA for 5 minutes at 37 °C. The cells were detached from the Transwell inserts by scraping with a pipette and collected in cold BM with 10% FBS to stop trypsinization. Cells were centrifuged (5 min, 350 *g*), the pellet was washed once with PBS and centrifuged again (5 min, 350 *g*). Pellets were resuspended in RNA lysis buffer (Qiagen) with 1% β-mercaptoethanol and lysates were stored at -80 °C until extraction. RNA extraction was performed using the Promega ReliaPrep miRNA Cell and Tissue Miniprep System (Z6211), according to the protocol provided. RNA quantity was determined by Nanodrop (Thermo Scientific).

### Gene Expression Analysis by Quantitative Reverse-Transcription PCR (RT-qPCR)

Complementary DNA (cDNA) was synthesized from 500 ng of template RNA using the SuperScript IV VILO Master Mix with ezDNase enzyme (Invitrogen, 11766050), according to the manufacturer’s protocol. Real-time quantitative PCR was performed using Taqman Fast Advanced Master Mix (Applied Biosystems, 4444556) with predesigned Taqman probes and run on a Viia 7 system (Applied Biosystems). Samples were run in duplicate and analyzed using the ∆∆Ct method with normalization to three reference genes (actin beta (*ACTB*), ribosomal protein lateral stalk subunit P0 (*RPLP0*), and glyceraldehyde-3-phosphate dehydrogenase (*GAPDH*)). Expression levels are presented as log2 fold changes compared to the non-inflamed control (CTRL). The genes included are related to the mucus layer (mucin 2 (*MUC2*), mucin 5ac (*MUC5AC*), mucin 5b (*MUC5B*)), epithelial barrier function (hypoxia-inducible factor 1 alpha subunit (*HIF1A*), tight junction protein 1 (*TJP1*), occludin (*OCLN*), claudin 1 (*CLDN1*)), and inflammation (interleukin 8 (*CXCL8*), interleukin 1 beta (*IL1B*), tumor necrosis factor (*TNF*), toll-like receptor 4 (*TLR4*)), and are listed in Table S2.

### Cytokine Measurements

Cytokine protein levels were quantified in both the apical and basolateral supernatant of organoid-derived monolayers using the V-PLEX Proinflammatory Panel 1 (human) kit (Meso Scale Diagnostics, Rockville, MD, USA), according to the manufacturer’s protocol. This kit provides assay-specific components for the quantitative determination of IFN-γ, IL-1β, IL-2, IL-4, IL-6, IL-8, IL-10, IL-12p70, IL-13, and TNF-α. Supernatant samples were diluted 2-fold. The plate was read on the MESO QuickPlex SQ 120MM instrument and protein concentrations were determined using the MSD Discovery Workbench 4.0.12 software.

### Statistics

Statistical analysis was performed with Graphpad Prism 10 software (San Diego, CA, USA). Normality of all data was checked using the Shapiro–Wilk test. TEER data of Caco-2/HT29-MTX cocultures were analyzed with a Kruskal–Wallis test, followed by Dunn’s multiple comparisons test. TEER, mRNA, and cytokine data of organoid-derived monolayers were analyzed with repeated-measures (RM) one-way ANOVA, followed by Šídák’s multiple comparisons test, for normally distributed data, and with a Friedman test, followed by Dunn’s multiple comparisons test for not normally distributed data. The significance level was set at 0.05.

## Results

### Carrageenan Does Not Affect the Permeability of Caco-2/HT29-MTX Cocultures

**Figure 2.**
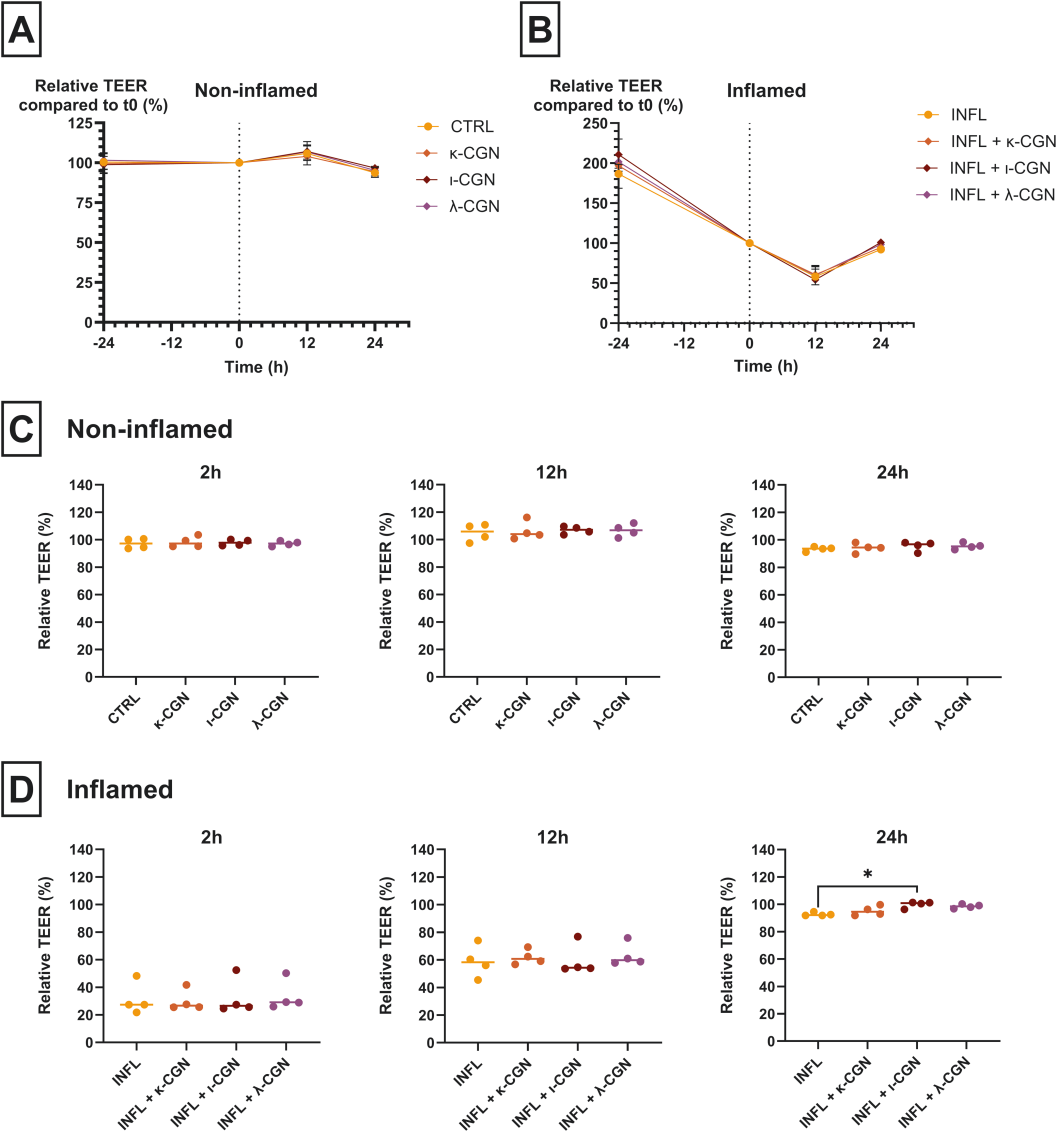
Carrageenan does not increase the permeability of Caco-2/HT29-MTX cocultures. A and B, Relative transepithelial electrical resistance (TEER) values (compared to t0) over time of non-inflamed (*n* = 4) (A) and inflamed (*n* = 4) (B) cell layers, stimulated with 100 µg mL^-1^ of κ-, ι-, or λ-carrageenan (CGN). C, Relative TEER of non-inflamed cell layers (*n* = 4) after 2, 4, and 24 hours of CGN stimulation. D, Relative TEER of inflamed cell layers (*n* = 4) after 2, 4, and 24 hours of CGN stimulation. Kruskal–Wallis test with Dunn’s multiple comparisons test, *P < .05. Abbreviations: CTRL, non-inflamed control; INFL, inflamed control.

Caco-2/HT29-MTX cocultures (*n* = 4) were stimulated with 100 µg mL^-1^ of three different types of dietary CGN (κ-, ι-, and λ-CGN) and their effect on the permeability of the monolayers was assessed by measuring TEER. In a non-inflamed condition, we observed no significant differences in TEER between monolayers simulated with κ-CGN (*P* > .999; Kruskal–Wallis test), ι-CGN (*P* > .999; Kruskal–Wallis test), or λ-CGN (*P* > .999; Kruskal–Wallis test) for 24 hours, compared to the control (CTRL) (Figure 2A and C).

Compared to the non-inflamed condition, TEER of inflamed monolayers decreased within the first 12 hours, but this was not statistically significant (2 hours: *P* = .100; 12 hours: *P* = .090; Kruskal–Wallis test). After 24 hours, the TEER of inflamed monolayers restored back to baseline (Figure 2B and D). Also, in inflamed conditions, treatment with CGN for 24 hours did not increase permeability of the cell layers, reflected by TEER, compared to the inflamed control (INFL). After 24 hours, we observed that monolayers stimulated with ι-CGN had significantly higher TEER values, compared to the inflamed control (*P* = .014; Kruskal–Wallis test). Also at a higher concentration (10 mg mL^-1^), CGN did not increase permeability of Caco-2/HT29-MTX cocultures, independent of the CGN type (Figure S1).

### κ-CGN Does Not Directly Affect the Permeability of Organoid-Derived Epithelial Monolayers from Patients with CD

We hypothesize that patients with IBD have an increased susceptibility for barrier disruption caused by external stimuli, such as dietary components. Therefore, we tested if the emulsifier κ-CGN, the most abundant subtype in food products, affected the permeability of organoid-derived monolayers from patients with CD.

**Figure 3.**
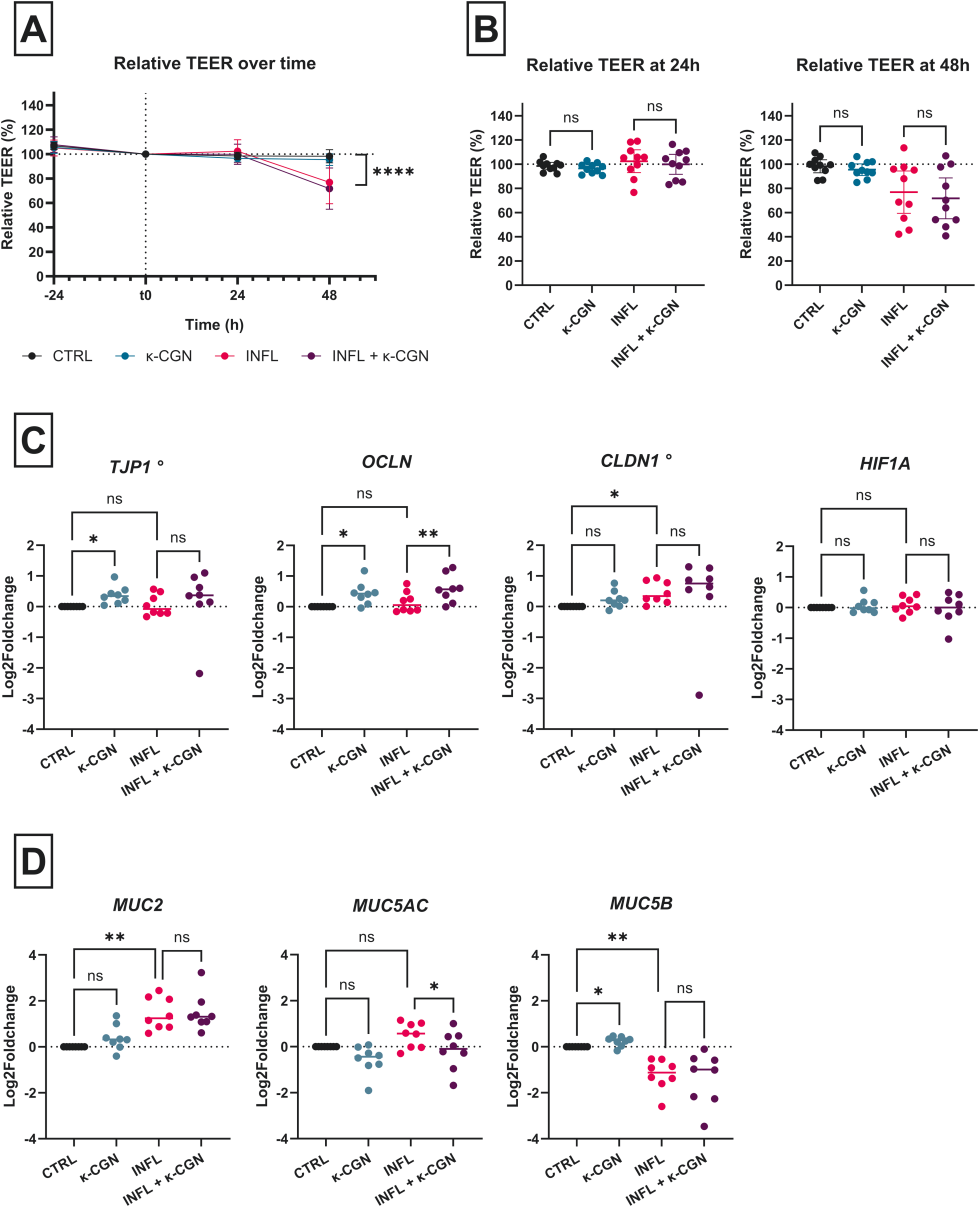
Kappa-carrageenan (κ-CGN) does not increase the permeability of organoid-derived monolayers from patients with Crohn’s disease. A, Relative transepithelial electrical resistance (TEER) values (compared to t0) over time. Transepithelial electrical resistance decreased significantly after 48 hours + 24 hours pre-stimulation with inflammatory stimuli (*P* < .001, Paired *t*-test). B, Stimulation with κ-CGN (100 µg mL^-1^) for 24 and 48 hours did not affect the relative TEER values of non-inflamed and inflamed epithelial monolayers (*n* = 10), compared to controls. C + D, κ-CGN induces minor changes in the expression of barrier-related genes. mRNA expression levels of tight junctions (C) and mucus proteins (D) were evaluated with RT-qPCR (*n* = 8). Expression levels are normalized against 3 housekeeping genes (ACTB, GPADH, RPLP0) and expressed as log2 fold changes, compared to CTRL. Results of repeated-measures one-way ANOVA followed by Šídák’s multiple comparisons test or (°) Friedman tests followed by Dunn’s multiple comparisons test are given (*P < .05, **P < .01, ***P < .001, ****P < .0001, ns, nonsignificant). Abbreviations: CTRL, non-inflamed control; INFL, inflamed control; κ-CGN, kappa-carrageenan.

As expected, exposure to inflammatory stimuli on the basolateral side resulted in a significant reduction of TEER after 48 hours (+24 hours pre-stimulation), compared to the non-inflamed conditions (*P* < .001; paired *t*-test) (Figure 3A). After 24 hours stimulation with κ-CGN, TEER did not significantly change in both the non-inflamed (*P* = .896; RM one-way ANOVA) and inflamed monolayers (*P* = .795; RM one-way ANOVA), compared to the control conditions. Also after 48 hours, exposure to κ-CGN did not significantly affect the TEER in neither non-inflamed (*P* = .943; RM one-way ANOVA) nor inflamed (*P* = .743; RM one-way ANOVA) conditions (Figure 3B).

### κ-CGN Induces Minor Changes in the Expression of Barrier-Related Genes

Organoid-derived monolayers from CD patients were stimulated with κ-CGN for 48 hours, after which RNA was extracted for RT-qPCR. Some slight changes in the expression of tight junction-related genes were detected after κ-CGN exposure (Figure 3C). The expression of *TJP1*, also known as *ZO1*, (*P* = .036; Friedman test) and *OCLN* (*P* = .037; RM one-way ANOVA) increased significantly after stimulation with κ-CGN in a non-inflamed condition, compared to the control. Also in the inflamed condition, the expression of *OCLN* increased upon κ-CGN stimulation (*P* = .001; RM one-way ANOVA). The expression of *CLDN1* was increased in the inflamed conditions (*P* = .020; Friedman test), compared to the non-inflamed monolayers, but was not affected by stimulation with κ-CGN. The expression of *HIF1A*, an upstream regulator of tight junction proteins, remained unaltered in all conditions.

Besides tight junctions, the mucus layer forms an important element of the intestinal barrier. Induction of inflammation resulted in a significant upregulation of *MUC2* (*P* = .002; RM one-way ANOVA) and a similar trend for *MUC5AC*, although not significant, was observed (*P* = .112; RM one-way ANOVA) (Figure 3D). *MUC5B* was significantly downregulated in the inflamed condition (*P* = .004; RM one-way ANOVA). Stimulation with κ-CGN resulted in a decrease of *MUC5AC* in inflamed monolayers (*P* = .031; RM one-way ANOVA) and an increase of *MUC5B* in non-inflamed monolayers (*P* = .049; RM one-way ANOVA).

### κ-CGN Exposure Causes Upregulation of Pro-Inflammatory Genes in Intestinal Epithelial Cells from CD Patients

**Figure 4.**
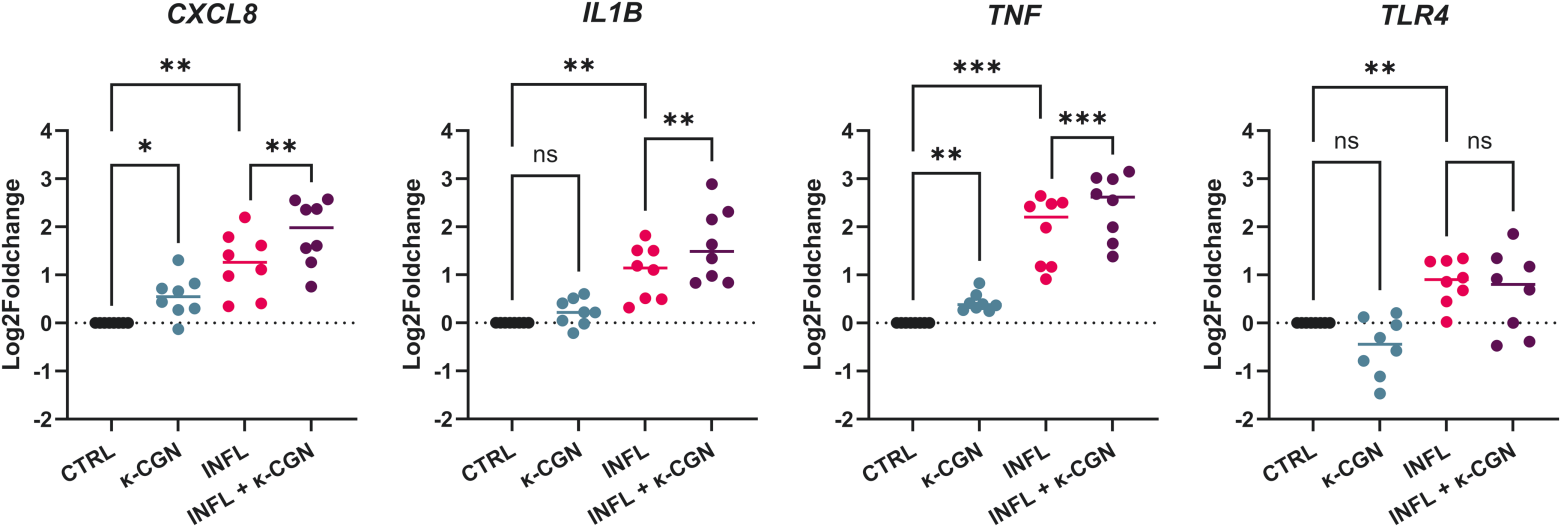
Kappa-carrageenan (κ-CGN) exposure caused upregulation of pro-inflammatory genes in organoid-derived monolayers from patients with Crohn’s disease. mRNA expression levels of inflammation-related genes were evaluated with RT-qPCR (*n* = 8). Expression levels are normalized against 3 housekeeping genes (ACTB, GPADH, RPLP0) and expressed as log2 fold changes, compared to CTRL. Results of repeated-measures one-way ANOVA followed by Šídák’s multiple comparisons test are given (*P < .05, **P < .01, ***P < .001, ns = non-significant). Abbreviations: CTRL, non-inflamed control; INFL, inflamed control.

To investigate if κ-CGN can induce or promote intestinal inflammation, we analyzed the expression of pro-inflammatory genes (*CXCL8, IL1B,* and *TNF*) in organoid-derived monolayers from CD patients, stimulated with the emulsifier for 48 hours (Figure 4). As expected, inflamed monolayers had a significantly higher expression of *CXCL8* (*P* = .003; RM one-way ANOVA), *IL1B* (*P* = .003; RM one-way ANOVA), and *TNF* (*P* < .001; RM one-way ANOVA), compared to the non-inflamed condition. When exposing non-inflamed monolayers to κ-CGN, the expression of *CXCL8* (*P* = .026, RM one-way ANOVA) and TNF (*P* = .001; RM one-way ANOVA) increased significantly. Interestingly, this pro-inflammatory effect of κ-CGN was even more pronounced when inflammation was already present, with increased expression levels of all pro-inflammatory markers upon κ-CGN stimulation (*CXCL8*: *P* = .010; *IL-1b*: *P* = .009; *TNF*: *P* = .001; RM one-way ANOVA).

Previous studies suggested that CGN can induce IL-8 secretion by binding to toll-like receptor 4 (TLR4).^12,28^ Therefore, we evaluated the gene expression of *TLR4* upon κ-CGN stimulation (Figure 4). In an inflamed state, *TLR4* was significantly upregulated compared to the non-inflamed control (*P* = .004; RM one-way ANOVA). However, κ-CGN had no significant effect on the expression levels of *TLR4* (non-inflamed: *P* = .149; Inflamed: *P* = .622; RM one-way ANOVA).

### κ-CGN Alters Cytokine Release of Intestinal Epithelial Cells from Patients with CD

**Figure 5.**
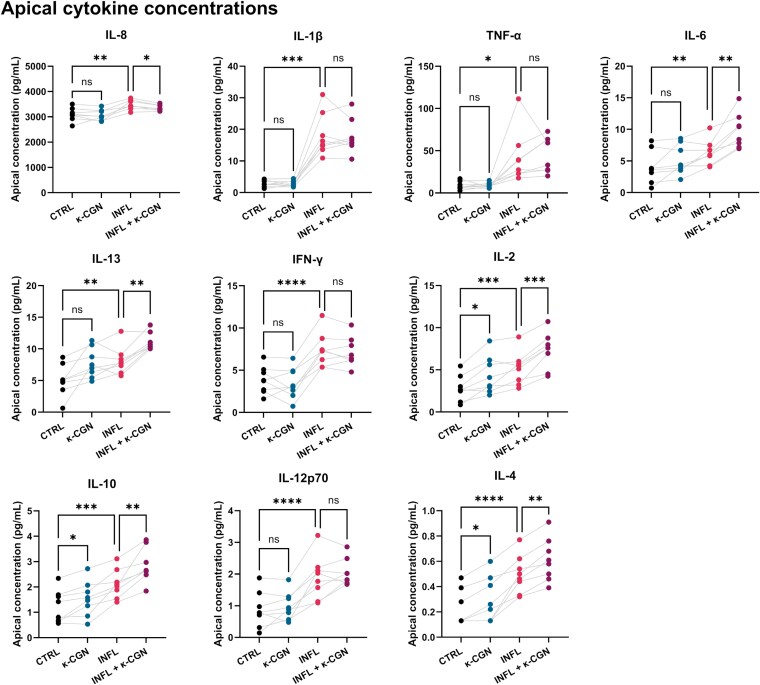
Kappa-carrageenan (κ-CGN) induces changes in the apical cytokine release of intestinal epithelial cells from Crohn’s disease (CD) patients. The level of pro-inflammatory cytokines was quantified in the apical supernatant after stimulating non-inflamed and inflamed epithelial monolayers from CD patients with κ-CGN for 48 hours (*n* = 8). Results of repeated-measures one-way ANOVA followed by Šídák’s multiple comparisons test are given (*P < .05, **P < .01, ***P < .001, ****P < .0001, ns = non-significant). Abbreviations: CTRL, non-inflamed control; INFL, inflamed control.

After exposure to the emulsifier κ-CGN for 48 hours, the apical and basolateral media of both non-inflamed and inflamed organoid-derived monolayers was collected for quantification of ten inflammation-related cytokines. The induction of inflammation in these monolayers was reflected by a significant increase in all evaluated cytokines both on the apical (Figure 5) and basolateral side (**Figure S2**), compared to the non-inflamed control. The respective *P* values are presented in **Table S3** and absolute cytokine concentrations are displayed in **Table S4**.

In non-inflamed monolayers, stimulation with κ-CGN resulted in significant higher levels of IL-2 (*P* = .017; RM one-way ANOVA), IL-10 (*P* = .049; RM one-way ANOVA), and IL-4 (*P* = .042; RM one-way ANOVA) on the apical side, compared to the control (CTRL) (**Figure 5**). Also, IL-13 was higher in the κ-CGN-stimulated monolayers, but this was borderline not significant (*P* = .055, RM one-way ANOVA). In contrast, no differences were observed in the basolateral compartment (**Figure S2**).

In inflamed monolayers, κ-CGN caused an increase in IL-6 (*P* = .006; RM one-way ANOVA), IL-13 (*P* = .003; RM one-way ANOVA), IL-2 (*P* < .001; RM one-way ANOVA), IL-10 (*P* = .007; RM one-way ANOVA) and IL-4 (*P* = .001; RM one-way ANOVA) in the apical media, compared to the inflamed control (INFL) (**Figure 5**). On the basolateral side, a significantly higher concentration of IL-4 (*P* = .011; RM one-way ANOVA) and a trend towards a higher concentration of IL-13 (*P* = .054; RM one-way ANOVA) was observed when inflamed monolayers were stimulated with κ-CGN, compared to the inflamed control (**Figure S2**). The apical and basolateral concentration of IL-8 was significantly lower in the inflamed monolayers stimulated with κ-CGN (apical: *P* = .022; basolateral: *P* < .001; RM one-way ANOVA).

## Discussion

The prevalence of IBD is increasing worldwide and the role of dietary factors in its pathogenesis is getting more and more recognized.^29^ Ultra-processed food consumption was found to be associated with an increased risk of developing IBD, and the need for IBD-related surgery.^8,30^ However, which exact components are responsible for this association and the underlying mechanisms are still unknown. Dietary emulsifiers have gained more attention over the years, as they have been shown to induce dysbiosis and intestinal inflammation in animal models and increased permeability in cell lines.^6,31,32^ The intestinal epithelium serves as a barrier between the gut microbiota and the immune cell compartment, carrying out an essential role in intestinal homeostasis.^33^ Intestinal barrier disruption is thought to be one of the first events in the pathogenesis of IBD, causing translocation of the microbiota and its products over the epithelium with subsequent activation of the immune system.^34^ Identifying compounds that influence the epithelial barrier is therefore of great interest. In this study, we showed that exposure to dietary κ-CGN for 48 hours induced pro-inflammatory changes when using organoid-derived monolayers from patients with CD, indicating a potential role in the development and exacerbation of intestinal inflammation. As far as we know, this is the first study using patient-derived intestinal organoids, which maintain disease-specific characteristics and the genetic background of the donor,^35,36^ to clarify the safety of CGN.

Stimulation with κ-CGN, the most frequently used subtype in food products, caused an increase in the gene expression of *CXCL8*, better known as *IL8*, *IL1B*, and *TNF*, both in non-inflamed and inflamed monolayers, but this was not confirmed at the protein level. Other pro-inflammatory cytokines were increasingly secreted when non-inflamed monolayers were stimulated with κ-CGN, including IL-2 and IL-4. In an inflamed condition, κ-CGN had a stronger effect on cytokine release, with an increase of IL-6, IL-13, IL-2, and IL-4, suggesting that CGN has the potential to maintain or even amplify intestinal inflammation during active IBD. Surprisingly, we found a lower concentration of IL-8, a potent attractant of immune cells, upon κ-CGN stimulation, compared to the inflamed control.

Similarly, an *in vitro* study with HT-29 cells showed that κ-CGN only induced IL-8 secretion in the presence of LPS.^37^ It could thus be possible that an external factor, such as LPS, is needed for CGN to induce IL-8 release, but this was not further investigated in our study. Studies conducted by the group of Tobacman et al. showed that dietary CGN induced IL-8 secretion by intestinal epithelial cells through activation of the NF-kB signaling pathway and TLR4 was identified as the responsible receptor.^12,28^ We evaluated if κ-CGN influenced the expression of TLR4 in organoid-derived monolayers, but did not detect significant differences.

Next, we studied if different CGN subtypes increased epithelial permeability, as reported by previous studies.^13,14^ No changes in the permeability of non-inflamed and inflamed Caco-2/HT29-MTX cocultures were observed after treating the cells with κ-, ι-, or λ-CGN for 24 hours. Also, when stimulating organoid-derived monolayers from CD patients with κ-CGN for 48 hours, no significant changes in permeability were observed, regardless of inflammatory state. In line, McKim et al. showed no effect on permeability when stimulating Caco-2 cells with CGN, at the same concentration range as our study.^16^ On gene expression level, we did detect slight alterations of tight-junction-related genes after stimulating organoid-derived monolayers with κ-CGN. The expression of *TJP1*, better known as *ZO*1, and *OCLN* were increased, suggesting a strengthening of the barrier. Interestingly, in mucosal tissue of IBD patients, an elevated expression of *CLDN1*, one of the barrier-strengthening claudins, has been observed.^38^ Similarly, we observed a higher expression of *CLDN1* in the inflamed monolayers. It has been proposed that the expression of *CLDN1* gets upregulated as a compensatory mechanism, but that the protein fails to localize at the tight junctions.^38^ In the same way, upregulation of *ZO1* and *OCLN* expression by κ-CGN could perhaps serve as a defense mechanism, without forming functional tight junctions, as this did not translate in higher TEER values of the monolayers.

In contrast to what we observed, several studies reported an increased permeability of epithelial models after a short stimulation with CGN. Choi et al. observed a reduction in TEER of HCT-8 epithelial cells, already after 20 minutes of CGN stimulation.^13^ The tight junction protein zonula occludens 1 (ZO-1) was reduced, explaining the increased permeability. Next, Jiang et al. also showed a significant reduction in TEER of Caco-2 cells, cocultured with THP-1 macrophage cells, after 4 hours of κ-CGN stimulation.^14^ Here, barrier disruption was attributed to the secretion of TNF-α by the THP-1 cells, suggesting that CGN not only influences epithelial cells but also the underlying immune cells. Indeed, also other studies showed that CGN is capable of activating macrophages and stimulating the secretion of TNF-α.^39^ The discrepancy with our findings might result from the presence/absence of other factors (eg, macrophages) or the use of different intestinal cell lines. Particularly, by combining the enterocyte-like Caco-2 cells with mucus-producing HT29-MTX cells, we ensured the presence of a mucus layer in our model.^40^ Because of this, the direct contact between CGN and the epithelial cells might have been limited and more resembling the physiological situation.

The intestinal epithelium is covered by a semipermeable mucus layer, which prevents direct contact between the gut microbiota and the epithelial cells. It has been recognized that defects in this mucus layer can be part of IBD development.^41^ We observed that the induction of inflammation altered the expression of *MUC2* and *MUC5B*, with a significant increased and decreased expression, respectively. Several dietary emulsifiers, such as polysorbate 80 and carboxymethylcellulose, have previously been shown to alter mucus layer thickness and structure.^31,42,43^ Here, we show a significantly lower expression of *MUC5AC* upon κ-CGN stimulation in inflamed epithelial monolayers of CD patients. *MUC5AC* was shown to be increased in active UC and to have a protective role in DSS-induced colitis.^44^ A reduction in this mucin during inflammation is therefore proposed to be detrimental, as *MUC5*-deficient mice displayed an elevated infiltration of neutrophils, and greater disease severity.^44^

It is important to note that this study has some limitations. First, these short stimulations may not sufficient to capture the full effect of a food additive that is likely to be ingested on a daily basis. Long-term exposure in animals has resulted in different outcomes. Weiner et al. observed no adverse effects from 90-day κ-CGN supplementation, while Shang et al. found that mice receiving CGN showed inflammatory infiltrates in the distal and proximal colon.^17,45^ Two human trials which investigated the effect of CGN supplementation on time to relapse in UC patients obtained conflicting results.^22,23^ In the study of Bhattacharyya et al., 3 out of 5 subjects who received CGN daily relapsed after 5 weeks, 32 weeks, and 42 weeks, while none of 7 patients receiving placebo relapsed, concluding that a CGN-free diet can prevent relapse in UC patients.^22^ In contrast, a cross-over study from Laatikainen et al. showed no impact of 7-day CGN supplementation.^23^ It is conceivable that a supplementation period of 7 days was not sufficient, which aligns with the fact that we only observed subtle changes after CGN exposures. When there is a repeated exposure for a long period of time, these small alterations might accumulate and eventually make the epithelium more prone for barrier disruption and inflammation.

Secondly, this study is lacking other relevant factors, besides the epithelial cells, CGN could possibly interact with. Indirect effects of CGN, through modulation of the gut microbiota or interaction with immune cells, could be of importance. Dietary CGN is not absorbed in the gastrointestinal tract, making it available for fermentation by the gut microbiota.^46^ For example, CGN was shown to reduce the abundance of *Akkermansia Muciniphila*, a bacterial strain which was found to have a therapeutic potential in IBD.^47^ Finally, CGN was tested as a single ingredient, which is an oversimplification of a real-word diet. In food products, different types of CGN are often combined and other additives can be present, which might enhance the small effects we observed. Possible synergistic effects combining different compounds should be explored in further experiments.

In the future, the interaction between CGN and the gut microbiota, and how this interaction is influencing epithelial barrier function and inflammation, should be further studied. In addition, the effects of long-term exposure should be evaluated in human trials, with a focus on outcomes related to inflammation and intestinal permeability. Fortunately, the first trials in this area are already underway (eg, NCT06552156).

In conclusion, we showed that dietary CGN increased the expression of pro-inflammatory markers and altered cytokine secretion by organoid-derived epithelial monolayers from CD patients, without affecting permeability. This indicates that dietary CGN is capable of inducing changes in the inflammatory profile of intestinal epithelial cells, but that these changes might be too small to cause an acute effect. However, during long-term exposure to CGN, as happens in daily life, these alterations could accumulate and could eventually lead to barrier disruption or inflammation. We hypothesize that chronic exposure to CGN primes the intestinal epithelium to be more prone for barrier disruption and developing inflammation, but that repeated exposure or the presence of an additional trigger, such as changes in microbiota composition, is needed. In addition, we observed stronger pro-inflammatory effects when inflammation was already present, as during active IBD, indicating that CGN restriction can be beneficial in patients with active disease. Overall, the results of this study support the adherence to a diet low in processed foods and emulsifiers for patients with IBD, especially during active inflammation.

## Supplementary data

Supplementary data is available at *Inflammatory Bowel Diseases* online.

izae306_suppl_Supplementary_Tables_S1-S4_Figures_S1-S2
