# Supplementary material for: Dietary Carrageenan Amplifies the Inflammatory Profile, but not Permeability, of Intestinal Epithelial Cells from Patients With Crohn’s Disease
Source: Inflamm Bowel Dis. 2024 Dec 24;31(5):1392–403. doi: 10.1093/ibd/izae306 (PMC12069985; doi:10.1093/ibd/izae306)
Supplement: izae306_suppl_Supplementary_Tables_S1-S4_Figures_S1-S2 [file izae306_suppl_supplementary_tables_s1-s4_figures_s1-s2.zip › Supplementary data.docx]

**SUPPLEMENTARY DATA**

Supplementary tables

**Supplementary Table 1**. Composition of Human Expansion Medium (HM). v/v: percentage volume of total end volume.

| **Component** | **Concentration** | **Manufacturer** | **Catalog nr.** |
| --- | --- | --- | --- |
| Wnt3A | 50% v/v | In house cell line | / |
| R-spondin | 20% v/v | In house cell line | / |
| Noggin | 10% v/v | In house cell line | / |
| EGF | 50 ng/ml | Life Technologies | PMG8043 |
| A83-01 | 500 nM | Tocris | 2939/10 |
| SB202190 | 10 µM | Sigma-Aldrich | S7067 |
| Nicotinamide | 10 mM | Sigma-Aldrich | N0636 |
| n-Acetylcysteine | 1.25 mM | Sigma-Aldrich | A9165 |
| B27 | 1x | Life Technologies | 17504044 |

**Supplementary Table 2.** Overview of Taqman probes for RT-qPCR

| **Gene symbol** | **Gene name** | **Taqman Gene Expression Assay ID** | **Category** |
| --- | --- | --- | --- |
| ***MUC2*** | Mucin 2 | Hs03005103_g1 | Mucus |
| ***MUC5AC*** | Mucin 5AC | Hs01365616_m1 | Mucus |
| ***MUC5B*** | Mucin 5B | Hs00861595_m1 | Mucus |
| ***HIF1A*** | Hypoxia inducible factor 1 alpha subunit | Hs00153153_m1 | Tight junctions |
| ***TJP1/ZO1*** | Tight junction protein 1 / zonula occludens 1 | Hs01551871_m1 | Tight junctions |
| ***OCLN*** | Occludin | Hs00170162_m1 | Tight junctions |
| ***CLDN1*** | Claudin 1 | Hs00221623_m1 | Tight junctions |
| ***CXCL8/IL8*** | C-X-C motif chemokine ligand 8 / interleukin 8 | Hs00174103_m1 | Inflammation |
| ***IL1B*** | Interleukin 1 beta | Hs01555410_m1 | Inflammation |
| ***TNF*** | Tumor necrosis factor | Hs00174128_m1 | Inflammation |
| ***TLR4*** | Toll like receptor 4 | Hs00152939_m1 | Inflammation |
| ***ACTB*** | actin beta | Hs01060665_g1 | Housekeeping |
| ***RPLP0*** | ribosomal protein lateral stalk subunit P0 | Hs99999902_m1 | Housekeeping |
| ***GAPDH*** | glyceraldehyde-3-phosphate dehydrogenase | Hs99999905_m1 | Housekeeping |

**Supplementary Table 3.** Adjusted p-values from cytokine quantification in the supernatant of organoid-derived monolayers after stimulation with κ-CGN for 48 hours (n = 8; Repeated measures one-way ANOVA). CTRL = non-inflamed control, INFL = inflamed control, CGN = carrageenan.

|  | Apical | | | Basolateral | | |
| --- | --- | --- | --- | --- | --- | --- |
|  | CTRL vs.  INFL | CTRL vs.  κ-CGN | INFL vs.  INFL + κ-CGN | CTRL vs.  INFL | CTRL vs.  κ-CGN | INFL vs.  INFL + κ-CGN |
| IL-8 | 0.0027 | 0.9504 | 0.0218 | <0.0001 | 0.2991 | 0.0003 |
| IL-1β | 0.0006 | >0.9999 | 0.9877 | <0.0001 | 0.0986 | 0.9879 |
| TNF-α | 0.0386 | 0.9831 | 0.9685 | <0.0001 | 0.4585 | 0.9090 |
| IL-6 | 0.0055 | 0.2860 | 0.0055 | <0.0001 | 0.7262 | 0.2327 |
| IL-13 | 0.0012 | 0.0547 | 0.0028 | <0.0001 | 0.7262 | 0.0537 |
| IFN-γ | <0.0001 | 0.8498 | 0.7988 | <0.0001 | 0.7262 | 0.9998 |
| IL-2 | 0.0005 | 0.0167 | 0.0008 | <0.0001 | 0.7262 | 0.4615 |
| IL-10 | 0.0001 | 0.0487 | 0.0074 | <0.0001 | 0.7262 | 0.9187 |
| IL-12p70 | <0.0001 | 0.6687 | 0.6521 | <0.0001 | NA | 0.0771 |
| IL-4 | <0.0001 | 0.0417 | 0.0011 | <0.0001 | NA | 0.0114 |

**Supplementary Table 4.** Absolute cytokine concentrations (pg/ml) in the supernatant of organoid-derived monolayers after stimulation with κ-CGN for 48 hours (n = 8). Concentrations are shown as mean ± standard deviation. CTRL = non-inflamed control, INFL = inflamed control, CGN = carrageenan.

|  | Apical (pg/ml) | | | |
| --- | --- | --- | --- | --- |
|  | **CTRL** | **κ-CGN** | **INFL** | **INFL + κ-CGN** |
| IL-8 | 3096 ± 256 | 3066 ± 209 | 3475 ± 185 | 3354 ± 113 |
| IL-1β | 2.84 ± 1.05 | 2.83 ± 0.90 | 18.13 ± 6.69 | 17.75 ± 5.40 |
| TNF-α | 8.76 ± 4.65 | 9.33 ± 2.93 | 42.13 ± 30.60 | 45.81 ± 20.92 |
| IL-6 | 4.02 ± 2.56 | 5.17 ± 2.33 | 6.41 ± 1.95 | 9.76 ± 2.72 |
| IL-13 | 5.04 ± 2.46 | 7.72 ± 2.35 | 8.14 ± 2.16 | 11.13 ± 1.36 |
| IFN-γ | 3.84 ± 1.59 | 3.47 ± 1.82 | 7.40 ± 2.00 | 7.15 ± 1.74 |
| IL-2 | 2.81 ± 1.50 | 4.35 ± 2.21 | 5.13 ± 1.93 | 7.30 ± 2.13 |
| IL-10 | 1.22 ± 0.64 | 1.54 ± 0.69 | 2.12 ± 0.56 | 2.86 ± 0.67 |
| IL-12p70 | 0.87 ± 0.56 | 1.00 ± 0.44 | 1.89 ± 0.68 | 2.05 ± 0.42 |
| IL-4 | 0.22 ± 0.14 | 0.29 ± 0.18 | 0.50 ± 0.15 | 0.61 ± 0.17 |
|  | **Basolateral (pg/ml)** | | | |
|  | **CTRL** | **κ-CGN** | **INFL** | **INFL + κ-CGN** |
| IL-8 | 260.5 ± 84.6 | 291.7 ± 107.4 | 3135 ± 289 | 2961 ± 291 |
| IL-1β | 0.50 ± 0.40 | 0.18 ± 0.16 | 3139 ± 231 | 3127 ± 227 |
| TNF-α | 2.38 ± 3.73 | 0.61 ± 0.55 | 5215 ± 134 | 5202 ± 116 |
| IL-6 | 0.75 ± 0.05 | 0.88 ± 0.43 | 10.91 ± 2.79 | 13.68 ± 3.70 |
| IL-13 | 0.75 ± 0.36 | 0.93 ± 0.88 | 16.38 ± 3.13 | 21.15 ± 2.74 |
| IFN-γ | 0.83 ± 0.28 | 1.06 ± 0.94 | 16.82 ± 2.76 | 16.87 ± 3.41 |
| IL-2 | 0.48 ± 0.27 | 0.48 ± 0.25 | 9.48 ± 1.76 | 8.99 ± 2.28 |
| IL-10 | 0.06 ± 0.06 | 0.09 ± 0.13 | 5.23 ± 0.95 | 5.00 ± 0.70 |
| IL-12p70 | 0.14 ± 0.00 | 0.14 ± 0.00 | 4.63 ± 0.70 | 5.11 ± 0.79 |
| IL-4 | 0.13 ± 0.00 | 0.13 ± 0.00 | 0.97 ± 0.18 | 1.09 ± 0.25 |

Supplementary figures


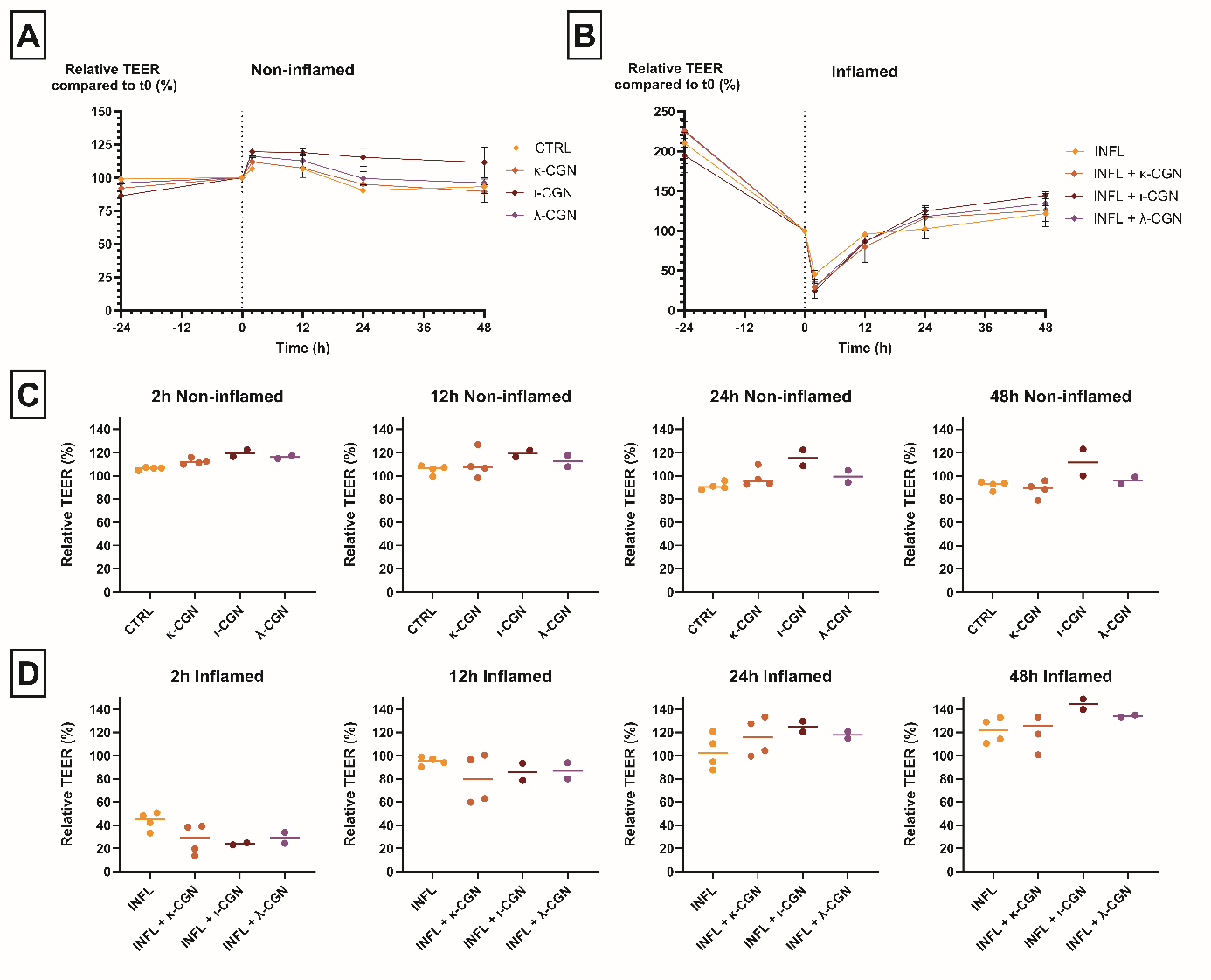


**Supplementary figure 1.** Carrageenan does not increase the permeability of a Caco-2/HT29-MTX coculture. **(A-B)** Relative TEER values (compared to t0) over time of non-inflamed (n = 4 technical replicates for CTRL, κ-CGN ; n = 2 technical replicates for ι-CGN, λ-CGN) (A) and inflamed (n = 4 technical replicates for INFL, INFL + κ-CGN ; n = 2 technical replicates for INFL + ι-CGN, INFL + λ-CGN) (B) cell layers, stimulated with 10 mg/ml of κ-, ι-, or λ-CGN. **(C)** Relative TEER of non-inflamed cell layers after 2, 12, 24 and 48 hours of CGN stimulation. **(D)** Relative TEER of inflamed cell layers after 2, 12, 24 and 48 hours of CGN stimulation. Brown-Forsythe and Welch ANOVA, * p < 0.05. CTRL = non-inflamed control, INFL = inflamed control, CGN = carrageenan, TEER = transepithelial electrical resistance.


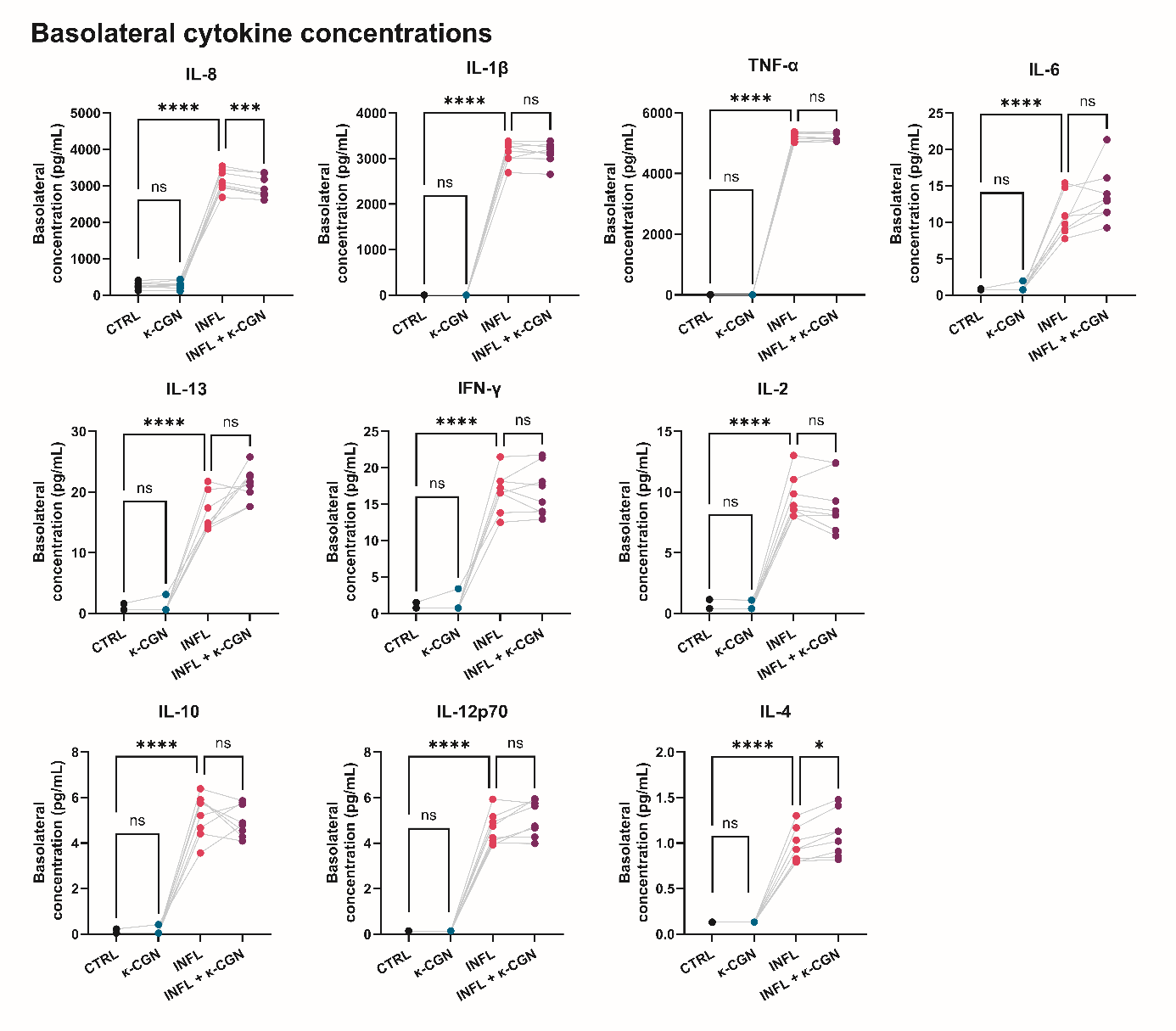


**Supplementary figure 2.** κ-CGN induces minor changes in the basolateral cytokine release of intestinal epithelial cells. The level of pro-inflammatory proteins was quantified in the basolateral supernatant after stimulating non-inflamed and inflamed epithelial monolayers from CD patients with κ-CGN for 48 hours. Results of RM one-way ANOVA followed by Šídák's multiple comparisons test are given (*p < 0.05, **p < 0.01, ***p < 0.001, ns = non-significant). CTRL = non-inflamed control, INFL = inflamed control, CGN = carrageenan.
